# Ventral root re-implantation is better than peripheral nerve transplantation for motoneuron survival and regeneration after spinal root avulsion injury

**DOI:** 10.1186/1471-2482-13-21

**Published:** 2013-06-24

**Authors:** Huanxing Su, Qiuju Yuan, Dajiang Qin, Xiaoying Yang, Wai-Man Wong, Kwok-Fai So, Wutian Wu

**Affiliations:** 1 State Key Laboratory of Quality Research in Chinese Medicine, Institute of Chinese Medical Sciences, University of Macau, Macao, China; 2 Department of Anatomy, Li Ka Shing Faculty of Medicine, The University of Hong Kong, 21 Sassoon Road, Pokfulam, Hong Kong SAR, China; 3 Research Center of Reproduction, Development and Growth, Li Ka Shing Faculty of Medicine, The University of Hong Kong, Pokfulam, Hong Kong SAR, China; 4 GHM Institute of CNS Regeneration, Jinan University, Guangzhou, China

**Keywords:** Avulsion, Peripheral nerve graft transplantation, Ventral root re-implantation, Motoneuron survival and regeneration

## Abstract

**Background:**

Peripheral nerve (PN) transplantation and ventral root implantation are the two common types of recovery operations to restore the connection between motoneurons and their target muscles after brachial plexus injury. Despite experience accumulated over the past decade, fundamental knowledge is still lacking concerning the efficacy of the two microsurgical interventions.

**Methods:**

Thirty-eight adult female Sprague–Dawley rats were divided into 5 groups. Immediately following root avulsion, animals in the first group (n = 8) and the second group (n = 8) received PN graft and ventral root implantation respectively. The third group (n = 8) and the fourth group (n = 8) received PN graft and ventral root implantation respectively at one week after root avulsion. The fifth group received root avulsion only as control (n = 6). The survival and axonal regeneration of severed motoneurons were investigated at 6 weeks post-implantation.

**Results:**

Re-implantation of ventral roots, both immediately after root avulsion and in delay, significantly increased the survival and regeneration of motoneurons in the avulsed segment of the spinal cord as compared with PN graft transplantation.

**Conclusions:**

The ventral root re-implantation is a better surgical repairing procedure than PN graft transplantation for brachial plexus injury because of its easier manipulation for re-implanting avulsed ventral roots to the preferred site, less possibility of causing additional damage and better effects on motoneuron survival and axonal regeneration.

## Background

PN grafts have been frequently used to restore the connectivity of peripheral targets with the spinal cord after avulsion injury to the brachial plexus [1,2]. The scar tissue containing astrocytic processes and numerous collagen fibers and the formation of the neuroma of avulsed spinal roots at the CNS–PNS border make it impossible to re-attach them directly to the spinal cord [3,4]. Thus, a PN graft is often applied to bridge the gap between the spinal nerve and the spinal cord. Experimental settings using PN transplantation have demonstrated its capacity of inducing reinnervation and protecting avulsed motoneurons from degeneration in various animal models [5-11]. Clinical application of PN grafts by direct implantation into the spinal cord leads to reinnervation and functional recovery in the proximal muscles in patients with severe brachial plexus injury [12]. In most cases, PN grafts were implanted into the lateral white matter of the spinal cord via the dorsal approach, resulting in the change of axon outgrowth direction from ventrally to laterally. Although the insertion site had been optimized so as to minimize functional disorder of the spinal cord [13], implantation may inflict damage to a not-yet traumatized area of the spinal cord. These limitations may discourage the clinical application of PN transplantation in surgically treating brachial plexus injury.

Surgical replantation of avulsed ventral roots was demonstrated to be effective in rescuing motoneurons, inducing axonal regeneration into the re-implanted ventral roots, and even promoting functional reinnervation of peripheral targets in various animal models [14-19]. Nearly all these implantation studies inserted the avulsed ventral rootlets into the parenchyma of the spinal cord to stimulate regeneration, which may cause additional damage to the spinal cord and require more challenging surgical skills.

We have developed a new microsurgical technique to restore the connection by positioning the avulsed ventral root on the ventrolateral pial surface of the spinal cord instead of inserting the ventral rootlets into the parenchyma of the spinal cord [20,21]. Re-implantation of the avulsed ventral root by attachment to the pial surface of rat spinal cords, both immediately after root avulsion and in delay, has been shown to be capable of inducing axonal regeneration of severed motoneurons to reinnervate muscle targets, leading to recovery of hand functions [21,22]. These findings suggest that implantation of the ventral root directly into the parenchyma of the spinal cord is not essential. Re-implantation of the avulsed ventral roots on the ventrolateral pial surface of the spinal cord could be less technically demanding and more easily manipulated through the dorsal approach. It could be a useful model to study axonal regeneration from CNS to PNS, identify inhibitory molecules in the pathway along axonal extension, and study the target innervation after brachial plexus injury.

Despite experience accumulated over the past decade, fundamental knowledge is still lacking concerning the efficacy of these microsurgical interventions. In the present study we investigated the survival and axon regeneration of severed motoneurons in the two distinct implantation models through the dorsal approach, i.e. PN graft transplantation model and ventral root implantation model. The results of the study provide evidence that the superficial implantation of ventral roots has better effects on motoneuron survival and regeneration after spinal root avulsion and may have clinical application potential in treating brachial plexus injury.

## Methods

Thirty-eight adult female Sprague–Dawley rats (220-250 g) were used in the present study and divided into 5 groups. Animals in the first group and the second group received PN graft transplantation (n = 8) and ventral root implantation (n = 8) respectively immediately following root avulsion; animals in the third group and fourth group received PN graft transplantation (n = 8) and ventral root re-implantation (n = 8) respectively at 1 week after root avulsion. The fifth group received root avulsion only as control (n = 6). All surgical interventions and subsequent care and treatment were approved by the Committee on the Use of Live Animals for Teaching and Research of the University of Hong Kong. Animals were anesthetized with an intraperitoneal injection of ketamine (80 mg/kg) and xylazine (8 mg/kg). Root avulsion was performed as described in our previous publications [20,21]. Briefly, a dorsal hemi-laminectomy on the right side of the sixth cervical vertebra was carried out under aseptic conditions. The 7th cervical spinal roots (C7) were avulsed by traction with a fine hook under a surgical microscope. For PN graft transplantation, an autologous saphenous nerve about 20 mm in length was harvested and a myelotomy approximately 1 mm deep was performed at the lateral funiculus of the C7 spinal cord. The PN was implanted into the myelotomy groove and further secured in position by suturing the epineurium of proximal end with 11–0 suture on the pia mater. The distal end of the graft was implanted into a juxtaposed skeletal muscle. For ventral root re-implantation, the avulsed ventral root was re-positioned on the ventrolateral pial surface of the spinal cord via the dorsal approach. The avulsed dorsal root was anchored to the edge of the dura mater with a suture to fix the repositioned ventral root in place. All the animals were allowed to survive for 6 weeks after PN grafting or ventral root implantation. Three days before the end of the survival period, 0.5 μl of 3% FG was injected into the C7 spinal nerve or the PN graft at the point 10 mm from the spinal cord to label the regenerating neurons.

At the end of the survival period, the animals were killed with a lethal dose of sodium pentobarbital and perfused intracardially with 0.01 M PBS, followed by perfusion with 200–300 ml of fixative solution containing 4% paraformaldehyde in 0.1 M PB. Spinal cords were harvested and postfixed in fresh fixative solution overnight and subsequently placed in 30% sucrose-0.1 M PB at 4°C for 2–3 days. The C7 segment of the spinal cord was cut into 30 μm cross sections on a microtome (American Optical Company, NY, USA), mounted on the slides, protected by cover slips and examined under a fluorescence microscope to count FG-positive cells. Only labeled neurons with visible nuclei were counted. Then we quantified the surviving motoneurons on neutral red stained sections (one of every other cross section) according to a previously described method [23,24]. Only those nucleolated profiles apparently belonging to motoneurons were counted to avoid duplication. The number of motoneurons on the intact side would be expressed as 100% of the control value. The number of surviving motoneurons on the lesioned side was described quantitatively as percentages of the normal control number.

Statistical differences between two groups were determined by two-tailed Student’s *t* test. Multiple group comparisons were made by one-way ANOVA and Tukey post hoc test. Data were presented as mean ± SEM. The significance level was set to 0.05 for all comparisons.

## Results

**Figure 1:**
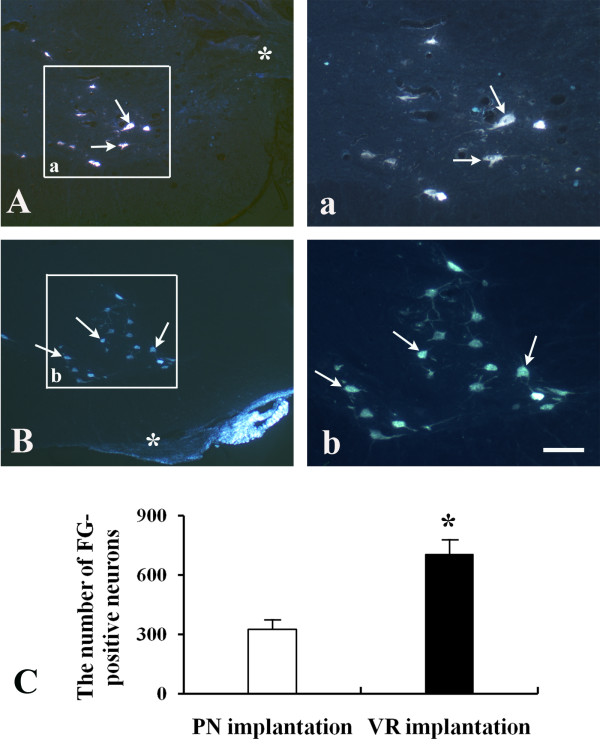
**Effects of PN graft transplantation and ventral root (VR) re-implantation on the axonal regeneration of avulsed motoneurons as revealed by retrograde FG-labeling at 6 weeks post-implantation. (A)** A representative micrograph of spinal cross sections showing FG-positive neurons (arrows) present in the ventral horn of the animals with PN graft transplantation (asterisk). **(B)** A representative micrograph of spinal cross sections showing FG-positive neurons (arrows) in the ventral horn of the animals with VR re-implantation (asterisk). **(a** and **b)** Micrographs made under higher magnification of the areas of interest in A and B, respectively. **(C)** The number of regenerating motoneurons in the VR re-implanted animals was significantly higher than that in the PN transplanted animals (*p < 0.001; scale bar: 200 μm in **A** and **B**; 80 μm in **a** and **b**).

Success of PN graft transplantation and ventral root implantation was confirmed by examining the integration of nerve with the host spinal cord during harvesting. All replanted PN grafts or ventral roots were found to be firmly connected with the spinal cord. Cross sections of the C7 segment further showed that the implanted PN (Figure 1A) and ventral root (Figure 1B) were nicely connected to the spinal cord. At 6 weeks post-implantation, retrograde labeling with FG revealed that approximately 325 ± 48.7 neurons in C7 spinal segment regenerated axons into the PN graft which was implanted immediately after root avulsion (Figure 1A and 1C). Notably, the number of regenerating neurons was markedly increased in the animals with ventral root implantation and about 703 ± 76.5 FG-positive neurons were detected in the ventral horn of these animals (P < 0.001 compared to PN-implanted animals, Figure 1B and 1C).

**Figure 2:**
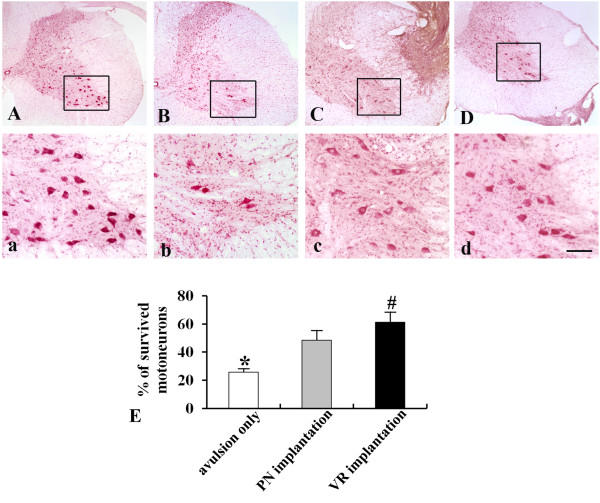
**Effects of PN graft transplantation and VR re-implantation on the survival of host motoneurons as revealed by neutral red staining at 6 weeks after root avulsion. (A)** Normal animals. **(B)** Animals receiving root avulsion only. **(C)** Animals receiving PN graft transplantation. **(D)** Animals receiving VR re-implantation. **(a**, **b**, **c**, and **d)** Micrographs made under higher magnification of the areas of interest in **A**, **B**, **C**, and **D**, respectively. **(E)** PN graft transplantation or VR re-implantation significantly increased the survival rate of motoneurons compared to controls. Furthermore, the survival rate of motoneurons in the animals receiving VR re-implantation was significantly higher than that seen in the animals receiving PN graft transplantation (*: p < 0.001 compared to PN or VR implantation; #: p < 0.05 compared to PN implantation; scale bar: 300 μm in **A**, **B**, **C** and **D**; 100 μm in **a**, **b**, **c** and **d**).

We then investigated the survival rate of motoneurons in these two implantation models. In the control group (root avulsion only), only 25.6 ± 2.8% of motoneurons survived in the ventral horn of the lesioned side 6 week after injury (Figure 2B and 2E) compared with the normal side (Figure 2A). Transplantation of a PN graft significantly increased the number of surviving motoneurons (48.3 ± 7.2%) compared with the control (P < 0.001; Figure 2C and 2E). The number of surviving motoneurons was further enhanced in the animals receiving ventral root implantation and 61.2 ± 7.3% of motoneurons survived at 6 weeks after injury, which is significantly higher than that in the animals receiving PN graft transplantation (P < 0.05, Figure 2D and 2E).

**Figure 3:**
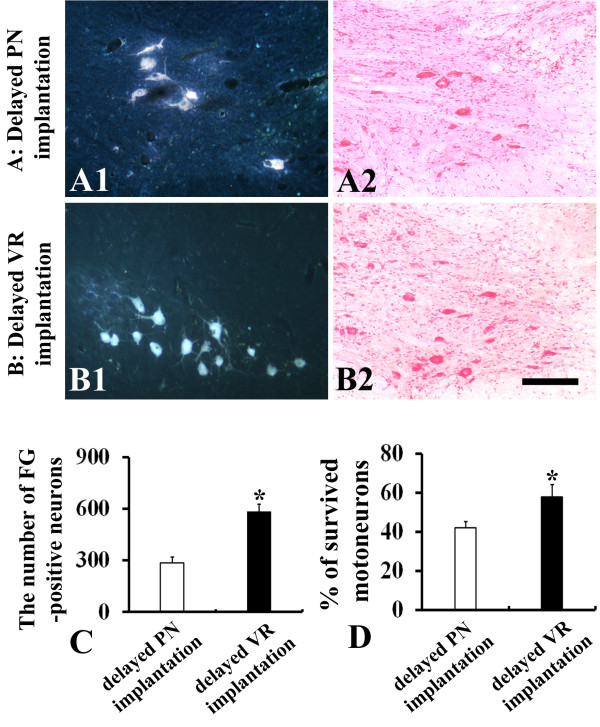
**Effects of delayed implantation of PN graft and VR on the survival and axonal regeneration of avulsed motoneurons at 6 weeks post-implantation. (A)** Animals with delayed implantation of PN graft. **(B)** Animals with delayed implantation of VR. **(C)** The number of regenerating neurons in the VR-implanted animals was significantly higher than that in the PN-implanted animals (*p < 0.001). **(D)** The survival rate of motoneurons in the VR-implanted animals was significantly higher than that in the PN-implanted animals (*p < 0.05). Scale bar: 200 μm in **A** and **B**.

A short time lag between the injury and the implantation surgery is recognized as a significant factor for root avulsion repair. Using the delayed implantation method, we further compared the effects of these two implantation models on the regeneration and survival of motoneurons. The PN graft or the avulsed ventral root was implanted at 1 week after root avulsion. At 6 weeks after implantation, the animals were killed. FG-labeling showed that delayed implantation of the PN graft induced 287 ± 32.3 neurons to regenerate their axons into the graft (Figure 3A1). The number of regenerating neurons was increased in the animals with delayed implantation of the ventral root and about 584 ± 42.5 FG-positive neurons were detected in the ventral horn of these animals (P < 0.001 compared to delayed PN-implanted animals, Figure 3B1 and 3C). Similarly, more surviving motoneurons were found in the ventral horn of the animals receiving delayed ventral root implantation and 57.9 ± 6.1% of motoneurons survived at 6 weeks after implantation, which is significantly higher than 42.7 ± 3.2% in the animals receiving delayed PN implantation (P < 0.05, Figure 3A2, B2 and 3D).

## Discussion

Ventral root re-implantation and PN graft transplantation are the two types of microsurgical interventions commonly used to restore the connection between motoneurons and their target muscles for the treatment of root avulsion injury. They can be performed via either a dorsal [5-8,14,25,26] or a ventral approach [10,27,28]. The dorsal approach is easier to perform in which only retraction of paraspinal muscles and hemi-laminectomy are needed to access the dura and the cord. The ventral approach has been used in several studies in order to implant avulsed rootlets into the original ventral exit zone [10,13,27,29,30]. However, it should be noted that it is more technically demanding for the implantation surgery via the ventral approach in which partial corporectomy is needed to expose the dura and avulsed area of the cord. In the present study we re-implanted the avulsed ventral roots on the ventrolateral pial surface of the spinal cord through the dorsal approach. Anatomical evaluations during harvesting spinal cords show that superficially replanted rootlets were firmly attached to the ventrolateral side of the cord, demonstrating that avulsed ventral rootlets can be repositioned to the preferred site (the ventral root outlet area) via the dorsal approach in the superficial implantation strategy.

After transplantation of PN grafts or implantation of avulsed ventral roots, significant numbers of motoneurons survived. Implanted PN grafts and ventral roots exert neuroprotective roles possibly through releasing neurotrophic substances that act on the perikarya of severed motoneurons and/or activate specific survival genes. The ventral exit zone possesses natural conduits connecting with motoneurons [31]. Repositioned ventral roots which are attached to the ventrolateral surface of the spinal cord may diffuse neurotrophic factors more efficiently than laterally implanted PN grafts, which do not connect with those natural conduits. Moreover, an interesting study reported that the ventral root preferentially supported motor axon regeneration since mRNA for pleiotrophin (PTN) and glial cell line-derived neurotrophic factor was upregulated to a greater degree in the ventral root [32]. These may account for the stronger effect of superficially implanted ventral roots on promoting motoneuron survival and regeneration compared to PN grafts. In addition, the distal-target environment between the two groups is different in the present study: the distal end of the PN graft was inserted in a juxtaposed muscle, while the distal part of avulsed ventral root was connected to the nerve. Whether the difference in the distal-target environment between the two groups affects motoneuron survival and regeneration needs to be further investigated in future studies.

Motoneurons in the ventral horn possess dendrites reaching the subpial surface as evidenced by intracellular injection experiments [33]. Following root avulsion, motoneurons respond with a changing polarity towards production of axons, sometimes even from the dendritic tree [34]. In addition, avulsion usually leaves tufts of the most proximal parts of roots attached to the spinal cord surface. All these may help injured motoneurons re-grow their axons into the implanted ventral roots on the surface of the spinal cord.

Root avulsion most frequently occurs in multitraumatized events and it is usually difficult to determine the exact location and degree of the injury. Therefore, delayed nerve repair has been advocated. However, avulsed spinal nerve roots have retracted in the delayed treatment, which reduces the length of root available for reconnection to the cord. PN transplantation has been utilized experimentally and clinically to bridge the gap resulting from spinal nerve retraction [9,12]. However, PN transplantation may cause additional damage to the spinal cord due to the insertion procedure. Harvesting the PN graft from patients also causes additional injury in another part of the body. The superficial implantation of avulsed ventral root in our study obviates these limitations associated with PN transplantation. Moreover, in the present study, we have demonstrated that avulsed ventral roots can be reconnected to the spinal cord after one week delay, and the repair can rescue more motoneurons and permit more surviving neurons to regenerate axons into the superficially implanted ventral root compared to the PN graft. The success of delayed implantation of avulsed ventral roots, though in only one week delay, raises the possibility of an alternative treatment for patients with avulsion injuries of the ventral roots.

## Conclusions

The ventral root re-implantation is a better surgical repairing procedure than PN graft transplantation for brachial plexus injury because of its easier manipulation for re-implanting avulsed ventral roots to the preferred site, less possibility of causing additional damage and better effects on motoneuron survival and axonal regeneration.

## Competing interests

The authors declare that they have no competing interests.

## Authors’ contributions

Conceived and designed the experiments: HS, KS, WW; Performed the experiments: QY, DQ, XY, WW; Analyzed the data: HS, KS, WW; Wrote the paper: HS, WW. All authors read and approved the final manuscript.

## Pre-publication history

The pre-publication history for this paper can be accessed here:

http://www.biomedcentral.com/1471-2482/13/21/prepub
